# 2019 WSES guidelines for the management of severe acute pancreatitis

**DOI:** 10.1186/s13017-019-0247-0

**Published:** 2019-06-13

**Authors:** Ari Leppäniemi, Matti Tolonen, Antonio Tarasconi, Helmut Segovia-Lohse, Emiliano Gamberini, Andrew W. Kirkpatrick, Chad G. Ball, Neil Parry, Massimo Sartelli, Daan Wolbrink, Harry van Goor, Gianluca Baiocchi, Luca Ansaloni, Walter Biffl, Federico Coccolini, Salomone Di Saverio, Yoram Kluger, Ernest Moore, Fausto Catena

**Affiliations:** 1 Abdominal Center, Helsinki University Hospital Meilahti, Haartmaninkatu 4, FI-00029 Helsinki,, Finland; 2 Department of Emergency Surgery, Parma Maggiore Hospital, Parma, Italy; 3 Hospital de Clinicas, Universidad Nacional de Asuncion, Asuncion, Paraguay; 4 Anesthesia and Intensive Care Medicine, Maurizio Bufalini Hospital, Cesena, Italy; 5 Foothills Medical Centre & the University of Calgary, Calgary, AB Canada; 6 London Health Sciences Centre, London, ON Canada; 7 Department of Surgery, Macerata Hospital, Macerata, Italy; 8 Radboud University Nijmegen, Nijmegen, The Netherlands; 9 Surgical Clinic, Department of Experimental and Clinical Sciences, University of Brescia, Brescia, Italy; 10 General, Emergency and Trauma Surgery Department, Bufalini hospital, Cesena, Italy; 11 Trauma and Acute Care Surgery, Scripps memorial Hospital, La Jolla, CA USA; 12 Cambridge University Hospitals NHS Foundation Trust, Cambridge, UK; 13 Division of General Surgery, Rambam Health Care Campus, Haifa, Israel; 14 Trauma Surgery, Denver Health Medical Center, Denver, CO USA

**Keywords:** Acute pancreatitis, Necrosectomy, Infected necrosis, Open abdomen, Consensus statement

## Abstract

Although most patients with acute pancreatitis have the mild form of the disease, about 20–30% develops a severe form, often associated with single or multiple organ dysfunction requiring intensive care. Identifying the severe form early is one of the major challenges in managing severe acute pancreatitis. Infection of the pancreatic and peripancreatic necrosis occurs in about 20–40% of patients with severe acute pancreatitis, and is associated with worsening organ dysfunctions. While most patients with sterile necrosis can be managed nonoperatively, patients with infected necrosis usually require an intervention that can be percutaneous, endoscopic, or open surgical. These guidelines present evidence-based international consensus statements on the management of severe acute pancreatitis from collaboration of a panel of experts meeting during the World Congress of Emergency Surgery in June 27–30, 2018 in Bertinoro, Italy. The main topics of these guidelines fall under the following topics: Diagnosis, Antibiotic treatment, Management in the Intensive Care Unit, Surgical and operative management, and Open abdomen.

## Introduction

Acute pancreatitis is an inflammatory condition of the pancreas most commonly caused by bile stones or excessive use of alcohol. In most patients, the disease takes a mild course, where moderate fluid resuscitation, management of pain and nausea, and early oral feeding result in rapid clinical improvement.

The severe form comprising about 20–30% of the patients is a life-threatening disease with hospital mortality rates of about 15% [1]. The most commonly used classification system for acute pancreatitis is the 2012 revision of the Atlanta classification and definitions based on international consensus [2]. This classification identifies two phases (early and late). Severity is classified as mild, moderate, or severe. The mild form (interstitial edematous pancreatitis) has no organ failure, local or system complications, and usually resolves in the first week. If there is transient (less than 48 h) organ failure, local complications or exacerbation of co-morbid disease, it is classified as moderate. Patients with persistent (more than 48 h) organ failure have the severe form of the disease.

Infection of the pancreatic and peripancreatic necrosis occurs in about 20–40% of patients with severe acute pancreatitis, and is associated with worsening organ dysfunctions. In a systematic review and meta-analysis totaling 6970 patients, the mortality rate in patients with infected necrosis and organ failure was 35.2% while concomitant sterile necrosis and organ failure was associated with a mortality of 19.8%. If the patients had infected necrosis without organ failure, the mortality was 1.4% [3].

According to the updated Atlanta classification 2012, the peripancreatic collections associated with necrosis are acute necrotic collection (ANC) and walled-off necrosis (WON) [2]. ANC is a collection seen during the first 4 weeks and containing variable amount of fluid and necrotic tissue involving the pancreatic parenchyma and/or peripancreatic tissues. WON is a mature, encapsulated collection of pancreatic and/or peripancreatic necrosis with a well-defined, enhancing inflammatory wall. The maturation takes usually 4 weeks or more after the onset of acute pancreatitis.

Currently, several trends in the management of severe acute pancreatitis have changed our clinical practices; early enteral feeding, selective role of prophylactic antibiotics, avoiding surgery in patients with sterile necrosis, more conservative approach to infected necrosis with delayed intervention, whether endoscopic or surgical, and management of biliary pancreatitis. The aim of these guidelines is to present evidence-based international consensus statements on the management of severe acute pancreatitis from collaboration of a panel of experts meeting during the World Congress of Emergency Surgery in June 27–30, 2018 in Bertinoro, Italy.

## Methods

These guidelines have been created by international collaboration and discussion among an expert panel of clinicians, practicing in the field of emergency surgery and managing patients with severe acute pancreatitis. These consensus guidelines have been facilitated by the World Society of Emergency Surgery, and are an update of the 2014 World Society of Emergency Surgery (WSES) position paper on this topic [4].

The statements are formulated and graded according to the Grading of Recommendations Assessment, Development and Evaluation (GRADE) hierarchy of evidence from Guyatt and colleagues [5], summarized in Table 1.

**Table 1:** Grading of Recommendations Assessment, Development and Evaluation (GRADE) hierarchy of evidence from Guyatt et al. [5]

Grade of recommendation	Clarity of risk/benefit	Quality of supporting evidence	Implications
1A			
Strong recommendation, high-quality evidence	Benefits clearly outweigh risk and burdens, or vice versa	RCTs without important limitations or overwhelming evidence from observational studies	Strong recommendation, applies to most patients in most circumstances without reservation
1B			
Strong recommendation, moderate-quality evidence	Benefits clearly outweigh risk and burdens, or vice versa	RCTs with important limitations (inconsistent results, methodological flaws, indirect analyses or imprecise conclusions) or exceptionally strong evidence from observational studies	Strong recommendation, applies to most patients in most circumstances without reservation
1C			
Strong recommendation, low-quality or very low-quality evidence	Benefits clearly outweigh risk and burdens, or vice versa	Observational studies or case series	Strong recommendation but subject to change when higher quality evidence becomes available
2A			
Weak recommendation, high-quality evidence	Benefits closely balanced with risks and burden	RCTs without important limitations or overwhelming evidence from observational studies	Weak recommendation, best action may differ depending on the patient, treatment circumstances, or social values
2B			
Weak recommendation, moderate-quality evidence	Benefits closely balanced with risks and burden	RCTs with important limitations (inconsistent results, methodological flaws, indirect or imprecise) or exceptionally strong evidence from observational studies	Weak recommendation, best action may differ depending on the patient, treatment circumstances, or social values
2C			
Weak recommendation, Low-quality or very low-quality evidence	Uncertainty in the estimates of benefits, risks, and burden; benefits, risk, and burden may be closely balanced	Observational studies or case series	Very weak recommendation; alternative treatments may be equally reasonable and merit consideration

For clarity, the statements and discussions have been divided into five topics: Diagnosis, Antibiotic treatment, Management in the Intensive Care Unit (ICU), Surgical and operative management, and Open abdomen.

## Results

### Diagnosis

Questions:

1. Which are the criteria to establish the diagnosis of severe acute pancreatitis?
2. What is the appropriate imaging work-up in case of suspected severe acute pancreatitis? What is the role of magnetic resonance imaging (MRI), computed tomography (CT) scan, ultrasound (US), endoscopic ultrasound (EUS), and other ancillary tests?
3. Which laboratory parameters should be considered in the diagnostic process?
4. How do different etiologies affect the diagnostic workup?
5. Which scores are indicated for risk assessment?
6. What is the timing and the suitable test for early follow-up imaging?

#### Statements (severity grading)

1. Severe acute pancreatitis is associated with persistent organ failure (cardiovascular, respiratory, and/or renal), and high mortality. Both new classification systems, Revised Atlanta Classification and Determinant-based Classification of Acute Pancreatitis Severity, are similar in establishing the diagnosis and severity of acute pancreatitis (1C).
2. Patients who have persistent organ failure with infected necrosis have the highest risk of death (1C).
3. Patients with organ failures should be admitted to an intensive care unit whenever possible (1C).

##### Discussion

Acute pancreatitis (AP) represents a disease characterized by acute inflammation of the pancreas and histologically acinar cell destruction [6]. The diagnosis of AP requires at least the presence of two of the three following criteria: (i) abdominal pain consistent with the disease, (ii) biochemical evidence of pancreatitis (serum amylase and/or lipase greater than three times the upper limit of normal), and (iii) characteristic findings from abdominal imaging [2].

Most patients (80–85%) will develop a mild disease course (self-limited, mortality < 1–3%), but around 20% will have a moderate or severe episode of AP, with a mortality rate from 13 to 35% [7, 8]. Thus, it is important to diagnose (or better predict) an episode of severe acute pancreatitis (SAP), and to identify the patients with high risk of developing complications.

During almost 20 years, the 1992 Atlanta Classification has been used, but some of the definitions and the classifications have been confusing [9]. In a revision of 447 articles, Bollen et al. found that alternative definitions of the 1992 Atlanta Classification were used in more than half of the studies, and that definitions are often used erroneously [9].

Important insights on the management of AP, better understanding of the pathophysiology of organ failure and necrotizing pancreatitis, improved diagnostic imaging, minimally invasive techniques, and studies showing that patients in the severe group of the 1992 Atlanta Classification comprise subgroups with very different outcomes, were indications that a more accurate classification is warranted.

In a review in 2004, Johnson et al. reported that persistent organ failure (POF) for more than 48 h in the first week is strongly associated with the risk of death or local complications [10]. They used a previous database of 290 patients with predicted SAP recruited from 78 hospitals through 18 centers in the UK, and also cited that resolution of organ failure within 48 h suggests a good prognosis.

A retrospective study of 759 patients with AP performed by the University of Edinburgh found that 25.4% of the patients with persistent systemic inflammatory response syndrome (SIRS) died, compared with 8% with transient SIRS and 0.7% without SIRS [11].

These and other studies showed that organ failure is central to the definition of SAP. If organ failure persists for more than 48 h, the patient is at high risk of death (one out of three) and a “severe” category can be established. Also, it is important to remind that a period of illness with a marked inflammatory response (SIRS) preceded the organ failure, and if SIRS is present, the patient is at risk of progression to organ failure, and every attempt should be made to restore normality as soon as possible [12].

Almost simultaneously in 2012, two new classifications systems of AP were published: Determinant-Based Classification of Acute Pancreatitis Severity (DBC) and the Revised Atlanta Classification 2012 (RAC) [2, 13]. The novel DBC was based on a global web-based survey and a dedicated international symposium with contributors from different disciplines: E-mail invitations were delivered to 528 pancreatologists from 55 countries, and 240 pancreatologists from 49 countries participated in the survey. During the 2011 World Congress of the International Association of Pancreatology (Kochi, India), around 100 participants discussed the proposed classification and tried to agree on the definitions [13].

The RAC was generated by an iterative, web-based consultation process incorporating responses from the members of 11 national and international pancreatic societies. Revisions were made in response to comments, and the web-based consultation was repeated three times. The final consensus was reviewed, and only statements based on published evidence were retained [2]. The RAC is a broader overview than DBC: in addition to severity classification, it provides a clear definition of diagnosing AP, highlights the onset of pain as an important reference point, and defines individual local complications as well as interstitial and necrotizing pancreatitis [2, 14]. The RAC has three categories: mild, moderately severe, and severe, according to organ failure and local or systemic complications. The DBC added a fourth category: critical, based on two main determinants of mortality: (peri)pancreatic necrosis and organ failure (Table 2).

**Table 2:** Definition of severity in acute pancreatitis

Revised Atlanta Classification (RAC)	Determinant-based classification (DBC
Mild acute pancreatitis (AP)	Mild AP
No organ failure	No organ failure AND
No local or systemic complications	No (peri)pancreatic necrosis
Moderately severe AP	Moderate AP
Transient organ failure (< 48 h)	Transient organ failure AND/OR
Local or systemic complications without persistent organ failure	Sterile (peri)pancreatic necrosis
Severe AP	Severe AP
Persistent single or multiple organ	Persistent organ failure OR
failure (> 48 h)	Infected (peri)pancreatic necrosis
	Critical AP
	Persistent organ failure AND
	Infected (peri)pancreatic necrosis

Subsequently, Bansal et al. in a cohort of 248 patients found that RAC and DBC are similar in ICU admission, need of percutaneous drainage, need for surgery, and in-hospital mortality. The critical category in DBC identified the most severe disease [15]. Nawaz et al. enrolled prospectively 256 patients, and assigned a severity category for all three classifications: RAC, DBC, and Atlanta 1992. They found that RAC and DBC severity categories accurately reflected clinical outcomes and were superior to Atlanta 1992 (evaluating mortality, ICU admission, ICU length of stay) [16].

Two years later, a retrospective study of 395 patients in China, with an overall 8.9% in-hospital mortality, found similar results. The authors found that all three classification systems (RAC, BDC, and Atlanta 1992) accurately classify the severity of AP. However, the RAC and the DBC performed better than the Atlanta 1992, and they were comparable in predicting long-term clinical prognosis, major complications, and clinical interventions [17].

Choi et al. studying 553 patients with AP admitted to a single center during the 7-year period, validated the RAC correlating well with clinical outcome, despite not considering infected necrosis. However, patients in the severe group and with infected necrosis (classified as critical in DBC) should be considered separately from those without it (the mortality rate increased fourfold: up to 32%) [18]. Another study analyzed 543 episodes of AP from 459 patients in a prospective cohort of patients. They found that the different categories of severity for each classification system were associated with statistically significant and clinically relevant differences in length of hospital stay, need for admission to the intensive care unit, nutritional support, invasive treatment, and in-hospital mortality. In addition, the direct comparison between categories of both classifications (after unifying the severe and critical category of the DBC) yielded no significant differences [19].

In general, patients with organ failure (accurately defined utilizing one of the established criteria or scoring systems) need an urgent transfer to an ICU. Accordingly, it may be unnecessary to transfer patients with transient organ failure to either a tertiary medical center or an ICU. Nevertheless, to confirm persistent organ failure, it needs to be documented for over 48 h.

#### Statements (imaging)

1. On admission, ultrasound (US) should be performed to determine the etiology of acute pancreatitis (biliary) (1C).
2. When doubt exists, computed tomography (CT) provides good evidence of the presence or absence of pancreatitis (1C).
3. All patients with severe acute pancreatitis need to be assessed with contrast-enhanced computed tomography (CE-CT) or magnetic resonance imaging (MRI). Optimal timing for first the CE-CT assessment is 72–96 h after onset of symptoms (1C).
4. Magnetic resonance cholangiopancreatography (MRCP) or endoscopic ultrasound should be considered to screen for occult common bile duct stones in patients with unknown etiology (1C).

##### Discussion

On admission, the etiology of AP should be determined, to project the need of definitive treatment (e.g., gallstone disease) and to avoid recurrence (e.g., alcohol intake, hypertriglyceridemia) [20]. The treatment and follow-up depend on the etiology of the AP. A transabdominal US should be performed on admission (to perform cholecystectomy for biliary pancreatitis when appropriate). Almost all the AP guidelines worldwide (based on revisions and meta-analyses) recommend performing US on admission or in the first 48 h [7, 8, 20–23].

In the majority of patients with AP, CT is not required [24]. The extension of the (peri)pancreatic necrosis may be detected with a contrast-enhanced CT (CECT) after 72 h from the onset of AP [20]. Concerns have been raised over post-contrast acute kidney injury (AKI). A recent meta-analysis with 28 observational studies and over 100,000 participants found no evidence to support the association of contrast with AKI, renal replacement therapy, or mortality [25]. However, there are no comparative studies in patients with severe acute pancreatitis or sepsis, and therefore, caution should be applied.

Early CT scan will not show necrotic/ischemic areas, and will not modify the clinical management during the first week of the illness. However, when the diagnosis is uncertain, CT should be considered, especially to rule out secondary perforation peritonitis or mesenteric ischemia. It also shows active hemorrhage and thrombosis associated with pancreatitis [21, 22].

CECT has been shown to yield an early overall detection rate of 90% with close to 100% sensitivity after 4 days for pancreatic necrosis [26]. Balthazar et al. established a CT severity index (Table 3) that graded pancreatitis based on the degree of inflammation, presence of fluid collections, and extent of necrosis: a higher score is associated with increased morbidity and mortality [26–28].

**Table 3:** CT Severity Index (Modified from: Balthazar EJ, Robinson DL, Megibow AJ, Ranson JH. Acute pancreatitis: value of CT in establishing prognosis. Radiology. 1990; 174:331–6 [27])

CT grade	Grade score	Definition
A	0	Normal pancreas
B	1	Pancreatic enlargement
C	2	Pancreatic inflammation and/or peripancreatic fat
D	3	Single peripancreatic fluid collection
E	4	≥ 2 fluid collections and/or retroperitoneal air
% of necrosis	Necrosis score	Definition
None	0	Uniform pancreatic enhancement
< 30%	2	Non-enhancement of region(s) of gland equivalent in size of pancreatic head
30–50%	4	Non-enhancement of 30–50% of the gland
> 50%	6	Non-enhancement of over 50% of the gland
CT Severity Index	Morbidity	Mortality
0–1	0	0
2–3	8%	3%
4–6	35%	6%
7–10	92%	17%

CT severity Index = grade score (0–4) + necrosis score (0–6)

CECT is the imaging modality of choice for diagnosis, staging, and detection of complications of acute pancreatitis, and has major roles in the evaluation of patients with known or suspected AP: (i) diagnosis, (ii) staging of the severity, and (iii) detection of complications, particularly the identification and quantification of (peri)pancreatic necrosis [20, 24, 26]. However, frequent repeat CT scans increase the total radiation dose and have limited effect in subsequent decision-making [29].

MRI is preferable to CECT in patients with allergy to iodinated contrast, in patients with renal impairment/insufficiency (unenhanced MRI), in young or pregnant patients to minimize radiation exposure in order to identify nonliquefied material (e.g., debris or necrotic tissue), but is less sensitive than CT for detecting gas in fluid collections [24, 26]. CT without contrast is an alternative for the first two patient groups, if MRI is not available.

When US does not show gallstones, sludge, or biliary obstruction and in the absence of cholangitis and/or abnormal liver function tests suggesting biliary obstruction, magnetic resonance cholangio-pancreatography (MRCP) or endoscopic ultrasound (EUS) rather than diagnostic endoscopic retrograde cholangiopancreatography (ERCP) should be used to screen for occult choledocholithiasis, if no other etiology can be established [20, 24]. In a retrospective cohort studying 221 patients, MRCP has a sensitivity of 97.98% and specificity of 84.4% for choledocholithiasis avoiding the need for invasive imaging in most patients with suspected choledocholithiasis [30].

#### Statements (diagnostic laboratory parameters)

1. The cut-off value of serum amylase and lipase is normally defined to be three times the upper limit.
2. C-reactive Protein level ≥ 150 mg/l at third day can be used as a prognostic factor for severe acute pancreatitis (2A).
3. Hematocrit > 44% represents an independent risk factor of pancreatic necrosis (1B).
4. Urea > 20 mg/dl represents itself as an independent predictor of mortality (2B).
5. Procalcitonin is the most sensitive laboratory test for detection of pancreatic infection, and low serum values appear to be strong negative predictors of infected necrosis (2A).
6. In the absence of gallstones or significant history of alcohol use, serum triglyceride and calcium levels should be measured. Serum triglyceride levels over 11.3 mmol/l (1000 mg/dl) indicate it as the etiology (2C).

##### Discussion

Serum pancreatic enzyme measurement is the “gold standard” for the diagnosis of AP [31]. In an episode of AP, amylase, lipase, elastase, and trypsin are released into the bloodstream at the same time but the clearance varies depending on the timing of blood sampling. Amylase is an enzyme secreted by the pancreas, and also salivary glands, small intestine, ovaries, adipose tissue, and skeletal muscles. There are two major isoforms of amylase: pancreatic and salivary, and the leading function is digestion of starch, glycogen, and related poly- and oligosaccharides, by hydrolysis [32]. In AP, serum amylase levels usually rise within 6 to 24 h, peak at 48 h, and decrease to normal or near normal levels over the next 3 to 7 days [23, 32, 33].

Lipase is another enzyme secreted by the pancreas. AP is the main reason for an increase in lipase, and many investigators emphasize that lipase is more specific, but can be found elevated also in non-pancreatic diseases such as renal disease, appendicitis, acute cholecystitis, chronic pancreatitis, bowel obstruction, etc. [23]. In AP, serum lipase remains elevated for a longer period than serum amylase. It rises within 4 to 8 h, peaks at 24 h, and decreases to normal or near normal levels over the next 8 to 14 days [32, 33].

Trypsinogen is the zymogen of the pancreatic enzyme trypsin. In AP, the serum and urinary concentrations of trypsinogen usually rise to high levels within a few hours and decrease in 3 days [32, 33].

Collectively, serum lipase is considered a more reliable diagnostic marker of AP than serum amylase. No single test shows optimal diagnostic accuracy, but most current guidelines and recommendations indicate that lipase should be preferred over total and p-amylase [32]. The main reasons supporting lipase over both types of amylase for the diagnosis of acute pancreatitis include higher sensitivity and larger diagnostic window [32]. A Cochrane revision with the aim to compare the diagnostic accuracy of different pancreatic enzymes in the diagnosis of AP showed a sensitivity and specificity of 72% and 93% for serum amylase, and 79% and 89% for serum lipase, respectively [33].

Chang et al. found in a meta-analysis including 13 studies that trypsinogen-2 dipstick test is a rapid and non-invasive bedside test with sensitivity 82% and specificity 94% for AP [34].

Numerous biomarkers have been studied as potential early predictors of the severity of AP so that treatment can be optimally tailored to prevent complications [34, 35]. At this moment, no laboratory test is practically available or consistently accurate to predict severity in patients with AP [23].

In the absence of gallstones or significant history of alcohol use, serum triglyceride should be measured and considered to be the etiology if the value is > 11.3 mmol/l (> 1000 mg/dl) [23].

Many textbooks consider the C-reactive protein (CRP) as the gold standard for disease severity assessment [36]. Using a cut-off value from 110 to 150 mg/l, the sensitivity and specificity ranged from 38 to 61%, and 89 to 90%, respectively, at the time of hospital admission [36]. The major drawback of CRP is that peak levels are reached only after 48 to 72 h.

In a prospective study of 175 patients divided into mild and non-mild acute pancreatitis according to the Atlanta classification, CRP and IL-6 combined demonstrated good discriminative capacity with an area under the curve of 0.803 [37].

Resistin is a newly identified peptide hormone, secreted specifically by adipocytes that can cause obesity and hypertriglyceridemia, due to its association with insulin resistance. Studies have revealed that resistin is also an important cytokine in inflammatory reactions, and in the regulation of other cytokines [38]. In a prospective observational study, resistin levels were better for predicting SAP than CRP or WBC levels on day 3, and better than CRP levels for predicting the development of necrosis [38]. A retrospective cohort study from data from 90 patients found that resistin has similar accuracy with the Acute Physiology and Chronic Health Evaluation II (APACHE II) score in predicting POF, and leptin has a weak correlation with POF [39].

Other laboratory findings used to characterize an episode of SAP are BUN > 20 mg/dl (> 7.14 mmol/l) or rising BUN, hematocrit (HCT) > 44% or rising HCT, lactate dehydrogenase (LDH), and procalcitonin for predicting infected necrosis in patients with confirmed pancreatic necrosis [36, 40–43]. A procalcitonin value of 3.8 ng/ml or higher within 96 h after onset of symptoms indicated a pancreatic necrosis with a sensitivity and specificity of 93% and 79% [36, 42]. Serum lactate level on admission predicts severe AP, death, and ICU admission, but should be considered suboptimal as a single marker [44].

#### Statements (diagnostics in idiopathic pancreatitis)

1. In idiopathic pancreatitis, biliary etiology should be ruled out with two ultrasound examinations, and if needed MRCP and/or endoscopic ultrasound EUS, to prevent recurrent pancreatitis (2B).

##### Discussion

Idiopathic AP is defined as pancreatitis with no etiology established after initial laboratory and imaging tests. In patients with idiopathic AP, at least two US examinations should be performed to rule out biliary etiology [31]. Following that, CE-CT and EUS, after the acute phase is over, are the next steps to assess microlithiasis, neoplasm, or chronic pancreatitis. If EUS is negative, MRI should be performed to identify morphologic abnormalities [31]. Laparoscopic cholecystectomy seems to prevent recurrent idiopathic acute pancreatitis; however, there is currently insufficient evidence to support this approach routinely [45].

#### Statement (risk scores)

1. There are no “gold standard” prognostic score for predicting severe acute pancreatitis. Probably the bedside index of severity of acute pancreatitis (BISAP) score is one of the most accurate and applicable in everyday clinical practice because of the simplicity and the capability to predict severity, death, and organ failure as well as the APACHE-II (very complex) and other scores (1B).

##### Discussion

Several scoring systems have been developed to predict SAP, but evidence on their predictive performance is variable [46, 47]. Currently, no systematic review has included studies assessing the accuracy of different clinical scoring systems used to predict severity and mortality in people with acute pancreatitis. Cochrane Database of Systematic Reviews is developing a protocol to synthesize studies evaluating the predictive accuracy of clinical scoring systems (measured on admission and up to 48 h following admission) [46].

Most prediction scores in AP have focused on death as an outcome. With the overall mortality declining over the past decades, it should be considered whether death should remain as the principal outcome to predict pancreatitis [48].

Another aspect is that more or less all severity scores take more than 24 h to stratify the patients, and probably that represent a loss of time in some critically ill patients [48]. A retrospective cohort study from UK conducted in 159 ICUs evaluating 2.462 patients admitted to ICU with SAP showed that 75% of the patients who required intensive care were transferred to the ICU within the first 72 h of admission to hospital, with a median time-to-transfer of 24 h after admission [49].

Over time, most scores were based on patient demographics, clinical features, laboratory parameters, or imaging modalities, and were assessed on admission or within 48 h: Ranson criteria (1974), Glasgow-Imrie score (1978), Acute Physiology and Chronic Health Evaluation II (APACHE II), Simplified Acute Physiology Score (SAPS II) (1984), Sequential Organ Failure Assessment (SOFA), CT severity index (CTSI), Bedside Index of Severity in Acute Pancreatitis (BISAP) score (2008), Japanese Severity Score [46].

The predictors (or potential predictors) present in almost all of the scoring systems mentioned above include age, organ failure or immunocompromise, previous history of chronic disease, temperature, blood pressure, pulse rate, respiratory rate, body mass index, consciousness level, presence of peritonitis, presence of acute renal failure, blood white cell count, blood hematocrit, blood platelet count, blood glucose, blood urea nitrogen, serum creatinine, serum aspartate transaminase, serum lactate dehydrogenase, serum calcium, serum electrolytes, serum bilirubin, plasma albumin, oxygen saturation, pH, and base deficit, and multiple imaging modalities principally CT.

The Apache II score evaluates the chronic health score and 12 physiologic measurement, but is not specific for AP, and is not designed for day to day evaluation in any patient. The advantages of this score are that it is a widely validated instrument and can be done at any time, but it has disadvantages; i.e., cumbersome and not all parameters are routinely collected [48]. In a study of 81 consecutive patients with AP, Thandassery et al. found that independent predictors of occurrence of infected necrosis were hypotension and APACHE II score at 24 h of hospital admission [50].

A study of 161 patients evaluated the assessment and comparison of the early predictability of various parameters most widely used in AP. They found the significant cutoff values for prediction of severe AP were Ranson ≥ 3, BISAP ≥ 2, APACHE-II ≥ 8, CTSI ≥ 3, and CRP at 24 h ≥ 21 mg/dl (> 210 mg/l). They concluded that different scoring systems showed similar predictive accuracy for severity of AP, but that APACHE-II demonstrated the highest accuracy for the prediction of SAP [51].

The PPV for the Ranson score ranges from 28.6 to 49% (sensitivity 75–87%, specificity 68–77.5%), for the Glasgow score from 59 to 66% (sensitivity 61–71%, specificity 88–89%), for the APACHE II score, 55.6% after 48 h (sensitivity 83.3%, specificity 91%), and for the APACHE-O score 54–80% (sensitivity 69–74%, specificity 86–90%). All these scores can only be assessed after 48 h, and thus do not enable risk stratification on admission. Despite their weaknesses, these scores are still useful to prove or exclude severe disease [31].

BISAP, a recently developed prognostic scoring system, has been proposed as a simple method for prediction of severe AP compared to traditional scoring systems. BISAP represent an acronym of the parameters evaluated in the score (Table 3) [48].

The BISAP score was derived using data from a population of 17,992 patients and validated on a population of 18,256 patients in the USA [52]. It has similar accuracy to the APACHE-II score for predicting death and is a very simplified scoring system that can be easily applied in the earliest phases. One of the key points of this study is that it was able to identify patients at increased risk of mortality prior to the onset of organ failure [52]. A retrospective analysis of 303 patients revealed that BISAP predicts severity, death, and especially organ failure (OF) in AP as well as APACHE-II does, and better than Ranson criteria, CT-severity index, CRP, hematocrit, and BMI. A BISAP score of two was a statistically significant cutoff value for the diagnosis of severe acute pancreatitis, organ failure, and mortality [53] (Table 4).

**Table 4:** Bedside index of severity of acute pancreatitis (BISAP) score [48]

BISAP: score one point for each of the following criteria	
Blood urea nitrogen level > 8.9 mmol/L	
Impaired mental status	
Systemic inflammatory response syndrome is present	
Age > 60 years	
Pleural effusion on radiography	

Multiple studies cite that BMI, obesity, and or overweight are independent risk factors for developing severe AP, local complications, or death [54, 55]. A study performed in two hospitals from Nanjing, China, using a cohort of 1073 patients to develop a new score and 326 patients to validate it, confirmed that changes in intra-abdominal pressure (IAP) and BMI were significantly associated with the severity of AP [46]. In addition, they found that the new modeling using BMI and changes in IAP has better sensitivity and specificity (77.6% and 82.6%) than APACHE-II (73.1% and 81.7%), BISAP (68.7% and 76.2%), CTSI (70.6% and 78.5%), and Ranson’s score (68.5% and 75.9%), respectively [55].

#### Statements (follow-up imaging)

1. In severe acute pancreatitis (computed tomography severity index ≥ 3), a follow-up CECT scan is indicated 7–10 days from the initial CT scan (1C).
2. Additional CE-CT scans are recommended only if clinical status deteriorates or fails to show continued improvement, or when invasive intervention is considered (1C).

##### Discussion

Patients with mild AP do not need a CT in the majority of cases. These patients will require further CT only if there is a change in the patient’s clinical status that suggests a new complication [20].

Routine follow-up CT (e.g., weekly or every 10 days) is advocated in several guidelines, but lack evidence to justify this practice. The vast majority of complications in a patient with AP/SAP can be suspected by clinical or laboratory assessment [20]. Therefore, in SAP, additional follow-up scans are recommended only if the patient’s clinical status deteriorates or fails to show continued improvement [21, 31].

The resolution of the CT manifestations of (peri)pancreatic inflammation virtually always lag behind the improving clinical status of the patient. Thus, if the patient shows an improving clinical status, additional follow-up scans during hospitalization are recommended only if the patient’s clinical status deteriorates or fails to show continued improvement.

### Antibiotic treatment

Questions

1. Which are the indications for an antimicrobial therapy in case of severe acute pancreatitis?
2. Is antibiotic prophylaxis effective in sterile severe acute pancreatitis?
3. What is the correct timing to introduce an antimicrobial therapy?
4. Which antimicrobial regimen should be used?
5. What is the correct duration of antimicrobial therapy?

#### Statement (prophylactic antibiotics)

1. Recent evidences have shown that prophylactic antibiotics in patients with acute pancreatitis are not associated with a significant decrease in mortality or morbidity. Thus, routine prophylactic antibiotics are no longer recommended for all patients with acute pancreatitis (1A).

##### Discussion

The use and efficacy of prophylactic antibiotic therapy in acute pancreatitis has long been a point of controversy. Prophylaxis refers to the administration of antibiotics in patients when no clinical infection is present with the intent to prevent pancreatic infection. Although early trials suggested that administration of antibiotics might prevent infectious complications in patients with sterile necrosis [56], subsequent, better-designed trials have consistently failed to confirm an advantage. Recent evidences have shown that prophylactic antibiotics in patients with acute pancreatitis are not associated with a significant decrease in mortality or morbidity [57–61]. Thus, routine prophylactic antibiotics for all patients with acute pancreatitis are no longer recommended.

#### Statement (infected necrosis and antibiotics)

1. Antibiotics are always recommended to treat infected severe acute pancreatitis. However the diagnosis is challenging due to the clinical picture that cannot be distinguished from other infectious complications or from the inflammatory status caused by acute pancreatitis (2A).
2. Serum measurements of procalcitonin (PCT) may be valuable in predicting the risk of developing infected pancreatic necrosis (1B).
3. A CT-guided fine-needle aspiration (FNA) for Gram stain and culture can confirm an infected severe acute pancreatitis and drive antibiotic therapy but is no longer in routine use (1B).

##### Discussion

Antibiotics are always recommended to treat infected acute pancreatitis. However, diagnosis of infected pancreatitis is challenging due to the clinical picture that cannot be distinguished from other infectious complications or from the inflammatory status caused by acute pancreatitis. The timing of infection in pancreatic necrosis is variable and unpredictable and peaks in the second to fourth week after the onset of pancreatitis. Clinical signs may be very sensitive yet are not specific enough [62, 63].

A limited number of smaller studies evaluated C-reactive protein (CRP). Conversely, PCT has been investigated as an effective predictor for the severity of acute pancreatitis and the risk of developing infected pancreatitis. PCT is the inactive 116 amino acid propeptide of the biologically active hormone calcitonin, which was first described to have significantly increased concentrations in patients with bacterial and fungal infections [64].

Several studies have demonstrated that serum measurements of PCT may be valuable in predicting the risk of developing infected pancreatic necrosis [65–68].

The diagnostic tool of choice remains CT-guided FNA of the pancreatic necrotic areas. A CT-guided FNA for Gram stain and culture can guide clinicians in choosing an appropriate individualized antibiotic regimen [69, 70]. However, because of the high rate of false negative findings, some centers have abandoned the routine use of FNA.

The presence of gas in the retroperitoneal area is considered indicative of infected pancreatitis in the context of severe acute pancreatitis, but it is only present in a limited number of patients [62].

#### Statement (type of antibiotics)

1. In patients with infected necrosis, antibiotics known to penetrate pancreatic necrosis should be used (1B).
2. In patients with infected necrosis, the spectrum of empirical antibiotic regimen should include both aerobic and anaerobic Gram-negative and Gram-positive microorganisms. Routine prophylactic administration of antifungal is not recommended in patients with infected acute pancreatitis, although *Candida* spp. are common in patients with infected pancreatic necrosis and indicate patients with a higher risk of mortality (1B).

##### Discussion

Aminoglycoside antibiotics (e.g., gentamicin and tobramycin) in standard intravenous dosages fail to penetrate into the pancreas in sufficient tissue concentrations to cover the minimal inhibitory concentration (MIC) of the bacteria that are commonly found in secondary pancreatic infections [71].

Acylureidopenicillins and third-generation cephalosporins have an intermediate penetration into pancreas tissue and are effective against gram-negative microorganisms and can cover the MIC for most gram-negative organisms found in pancreatic infections [72]. Among these antibiotics, only piperacillin/tazobactam is effective against gram-positive bacteria and anaerobes.

Quinolones (ciprofloxacin and moxifloxacin) and carbapenems both show good tissue penetration into the pancreas the additional benefit of excellent anaerobic coverage [73–76]. However, because of quinolones high rate of resistance worldwide, quinolones should be discouraged and used only in patients with allergy to beta-lactam agents. Carbapenems due to the spread of carbapenem resistant *Klebsiella pneumoniae* should be always optimized and should be used only in very critically ill patients.

Metronidazole, with its bactericidal spectrum focused almost exclusively against anaerobes, also shows good penetration into the pancreas.

Pathogenesis of secondary bacterial pancreatic infection is still debated. Pathogens can reach the pancreas through the hematogenous pathway, via the biliary system, ascending from the duodenum via the main pancreatic duct, or through transmural colonic migration via translocation of the colonic bacteria [77].

Most pathogens in pancreatic infection are gastrointestinal Gram-negative bacteria (*Escherichia coli*, *Proteus*, *Klebsiella pneumonia*), which occur via disruption of the intestinal flora and damage to the bowel mucosa. Impaired body defenses predispose to translocation of the gastrointestinal organisms and toxins with subsequent secondary pancreatic infection. However, Gram-positive bacteria (*Staphylococcus aureus*, *Streptococcus faecalis*, *Enterococcus*), anaerobes, and, occasionally, fungi have also been found [78].

Fungal infection is a serious complication of acute pancreatitis with an associated increase in morbidity and mortality [79]. *Candida albicans* is the most frequent organism encountered, followed by *Candida tropicalis* and *Candida krusei.* Although fungal infections complicating acute pancreatitis generally arise proportionately to the extent of pancreatic necrosis, there is not enough data to support the prevention of fungal infections and therefore is not recommended.

### Intensive care unit

Questions:

1. Which are the indications for intensive care unit (ICU) admission?
2. When is fluid resuscitation indicated and which fluid should be used? What is the optimal fluid infusion rate and response measurement for initial resuscitation? What is the preferred pharmacologic approach to persistent shock?
3. What is the correct approach for pain control?
4. Which are the indications for mechanical ventilation?
5. What is the medical approach to the abdominal compartment syndrome? What is the role of medications such as Gabexate Mesilate and somatostatin analogues?
6. Enteral nutrition: which are the indications, what type of nutrition should be used, and which is the best way to administer enteral nutrition?

#### Statement (monitoring)

1. Continuous vital signs monitoring in high dependency care unit is needed if organ dysfunction occurs. Persistent organ dysfunction or organ failure occurrence despite adequate fluid resuscitation is an indication for ICU admission (1C).

##### Discussion

The worldwide heterogeneity in intensive and intermediate care unit settings makes it difficult to define universal pathways. There is no single marker able to define the severity of the illness. Several scoring system should be used to assess the severity in a different phase, place, and patient.

Extensive fluid administration, adequate pain management with potentially harmful strategies, and organ function evaluation during initial treatment are the reason why continuous vital signs monitoring is crucial, whatever the setting is. Persistent organ dysfunction despite adequate fluid resuscitation needing specific organ support is usually delivered only in ICUs [11, 80].

#### Statement (fluid resuscitation)

1. Early fluid resuscitation is indicated to optimize tissue perfusion targets, without waiting for hemodynamic worsening. Fluid administration should be guided by frequent reassessment of the hemodynamic status, since fluid overload is known to have detrimental effects. Isotonic crystalloids are the preferred fluid (1B).

##### Discussion

The decrease in mortality observed over the last decade might be due to the prevention of pancreatic necrosis by maintenance of microcirculation due to more extensive fluid resuscitation. Data on the amount of fluid needed to prevent necrosis or to improve outcome are contradictory and the volume must be adjusted to the patient’s age, weight, and pre-existing renal and/or cardiac conditions [81].

Hematocrit, blood urea nitrogen, creatinine, and lactate are laboratory markers of volemia and adequate tissue perfusion, and should be monitored. Ringer’s lactate may be associated with anti-inflammatory effect, but the evidence for superiority of Ringer’s lactate vs. normal saline based on randomized trials is weak [82–84]. It could be better in correcting the potassium level. The value of early goal-directed therapy in patients with acute pancreatitis remains unknown [81, 85].

#### Statement (pain control)

1. No evidence or recommendation about any restriction in pain medication is available. Non-steroidal anti-inflammatory drugs (NSAID) should be avoided in acute kidney injury (AKI). Epidural analgesia should be an alternative or an agonist with intravenous analgesia, in a multimodal approach. Patient-controlled analgesia (PCA) should be integrated with every described strategy. (1C) Dilaudid is preferred over morphine or fentanyl in the non-intubated patient.

##### Discussion

Pain is the cardinal symptom of acute pancreatitis and its relief is a clinical priority. All patients with acute pancreatitis must receive some form of analgesia in the first 24 h of hospitalization in order not to compromise patient’s quality of life. In most institutions, dilaudid is preferred over morphine or fentanyl in the non-intubated patient. Epidural analgesia may be considered for those patients with severe and acute critical pancreatitis who require high doses of opioids for an extended period [63].

Despite some evidence from RCTs, there remains uncertainty about the preferred analgesic and the best method of administration. That is why the best current recommendation now is to adhere to the most current acute pain management guidelines in the perioperative setting [63].

#### Statement (mechanical ventilation)

1. Mechanical ventilation must be instituted if oxygen supply, even with high flow nasal oxygen, or continuous positive airway pressure became ineffective in correcting tachypnea and dyspnea. Both non-invasive and invasive techniques can be used, but invasive ventilation is mandatory when bronchial secretions clearance start to be ineffective and/or the patient is tiring of predicted to tire. Lung-protective strategies should be used when invasive ventilation is needed (1C).

##### Discussion

There are no issues for the management of respiratory failure specific to this topic. Oxygen supply, even with high flow or continuous positive pressure devices, could become insufficient in supporting respiratory failure. Different levels of tachypnea and dyspnea are only partially justified by hypoxia. Pain, possible intra-abdominal hypertension and pleural effusion, can induce these symptoms despite adequate arterial oxygenation. Increased systemic permeability could precipitate pulmonary edema after fluid resuscitation [86, 87].

#### Statement (increased intra-abdominal pressure)

1. Limitation of sedation, fluids, and vasoactive drugs to achieve resuscitative goals at lower normal limits is suggested. Deep sedation and paralysis can be necessary to limit intra-abdominal hypertension if all other nonoperative treatments including percutaneous drainage of intraperitoneal fluid are insufficient, before performing surgical abdominal decompression (1B)

##### Discussion

Increased systemic permeability induced by systemic inflammation and therapeutic attempts such as fluid resuscitation and vasoactive drugs are associated with gut failure and worsening of intra-abdominal pressure. Excessive sedation can further worsen gut dysfunction with subsequent increase in intra-abdominal pressure. Limiting “usual ICU medications” when side effects overcome benefits is crucial [88].

#### Statement (pharmacological treatment)

1. No specific pharmacological treatment except for organ support and nutrition should be given (1B).

##### Discussion

Despite a lot of research, no effective pharmacological treatment has been found [89].

#### Statement (enteral nutrition)

1. Enteral nutrition is recommended to prevent gut failure and infectious complications. Total parenteral nutrition (TPN) should be avoided but partial parenteral nutrition integration should be considered to reach caloric and protein requirements if enteral route is not completely tolerated. Both gastric and jejunal feeding can be delivered safely (1A).

##### Discussion

Enteral feeding maintains the gut mucosal barrier, prevents disruption, and prevents the translocation of bacteria that seed pancreatic necrosis. In most institutions, continuous infusion is preferred over cyclic or bolus administration. Enteral nutrition as compared with total parenteral nutrition decreases infectious complications, organ failure, and mortality [90]. In a multicenter, randomized study comparing early nasoenteric tube feeding within 24 h after randomization to an oral diet initiated 72 h after presentation to the emergency department with necrotizing pancreatitis, early nasoenteric feeding did not reduce the rate of infection or death. In the oral diet group, 69% of the patients tolerated an oral diet and did not require tube feeding [91].

### Surgical and operative management

Questions:

1. Which are the indications for emergent ERCP in case of severe acute pancreatitis?
2. Which is the correct operative/surgical strategy in severe acute pancreatitis?
3. Which are the indications for percutaneous/endoscopic drainage of pancreatic collections (i.e., sterile necrosis, infected necrosis, others)?
4. Which are the indications for surgical intervention?
5. What is the timing for surgery and what is the appropriate surgical strategy (i.e., laparoscopy vs. laparotomy, intraperitoneal vs. extraperitoneal, early vs. delayed)?
6. When is cholecystectomy recommended and what is the correct timing?

#### Statements (indications for emergent ERCP)

1. Routine ERCP with acute gallstone pancreatitis is not indicated (grade 1A).
2. ERCP in patients with acute gallstone pancreatitis and cholangitis is indicated (grade 1B).
3. ERCP in acute gallstone pancreatitis with common bile duct obstruction is indicated (grade 2B).
4. ERCP in patients with predicted severe acute gallstone pancreatitis without cholangitis or common bile duct obstruction cannot be recommended at this time (grade 2B).

##### Discussion

A systematic review of seven randomized controlled trials (RCT) comprising 757 participants found no evidence to support routine ERCP for all patients with acute gallstone pancreatitis (AGP) [92]. There was no evidence to suggest that the results were dependent on the predicted severity of AGP. However, concerns have been raised of study design limitations, lack of pooled sample size with predicted severe AGP, and ERCP timing and technique. In the same meta-analysis, among trials that included patients with cholangitis, the early routine ERCP significantly reduced mortality as well as local and systemic complications.

In patients with biliary obstruction, early routine ERCP was associated with a significant reduction in local complications and a non-significant trend toward reduction of systemic complications. In cases of predicted severe AGP, the guidelines are controversial [93]. This systematic review studied eight meta-analyses and 12 guidelines and concluded that consensus is lacking on routine ERCP with predicted severe AGP. An on-going RCT, the APEC trial, is designed to answer this question [94]. The recruitment has ended but the results have not yet been published.

#### Statement (indications for percutaneous/endoscopic drainage of pancreatic collections)

1. Clinical deterioration with signs or strong suspicion of infected necrotizing pancreatitis is an indication to perform intervention (percutaneous/endoscopic drainage)

After 4 weeks after the onset of the disease:

- On-going organ failure without sign of infected necrosis
- On-going gastric outlet, biliary, or intestinal obstruction due to a large walled off necrotic collection
- Disconnected duct syndrome
- Symptomatic or growing pseudocyst

After 8 weeks after the onset of the disease:

- On-going pain and/or discomfort

(grade 1C)

##### Discussion

The evidence of indications is based on understanding the natural course of the disease, mechanism-based reasoning, and non-randomized studies. Interventions for necrotizing pancreatitis should preferably be done when the necrosis has become walled-off, usually after 4 weeks after the onset of the disease [2].

Signs or strong suspicion of infected necrosis in a symptomatic patient requires intervention, although a small number of patients have been shown to recover with antibiotics only [1]. When a patient deteriorates a step-up approach starting with percutaneous or endoscopic drainage is indicated [20, 95–97].

A majority of patients with sterile necrotizing pancreatitis can be managed without interventions [1]. However, it should be noted that nearly half of patients operated due to on-going organ failure without signs of infected necrosis have a positive bacterial culture in the operative specimen [98]. Therefore, interventions should be considered when organ dysfunctions persist for more than 4 weeks.

Walled off necrotic collections or pseudocysts may cause symptoms and/or mechanical obstruction and if they do not resolve when inflammation ceases, a step up approach is indicated. A symptomatic disconnected pancreatic duct results in a peripancreatic collection and is an indication for interventions [99, 100].

#### Statements (indications for surgical intervention)

The following are indications for surgical intervention:

- As a continuum in a step-up approach after percutaneous/endoscopic procedure with the same indications
- Abdominal compartment syndrome
- Acute on-going bleeding when endovascular approach is unsuccessful
- Bowel ischaemia or acute necrotizing cholecystitis during acute pancreatitis
- Bowel fistula extending into a peripancreatic collection

(grade 1C)

##### Discussion

The evidence of indications is based on understanding the natural course of the disease, mechanism-based reasoning, and non-randomized studies. When percutaneous or endoscopic strategies fail to improve the patient, further surgical strategies should be considered. Abdominal compartment syndrome should first be managed by conservative methods [101]. Surgical decompression by laparostomy should be considered if conservative methods are insufficient [102].

Bleeding complications in acute severe pancreatitis may warrant surgical interventions if endovascular approach is unsuccessful. Bowel- and other extrapancreatic complications are relatively rare but may require surgical interventions.

#### Statement (timing of surgery)

1. Postponing surgical interventions for more than 4 weeks after the onset of the disease results in less mortality (2B).

##### Discussion

Early surgery was compared to late surgery in a recent systematic review and meta-analysis from the Eastern Association for the Surgery of Trauma [103]. The study consisted of nine studies, of which one was a randomized controlled study. Timing of operative interventions was compared in three different cut-offs (72 h, 12 days, and 30 days). In all cut-offs, late surgery resulted in a clear survival benefit. With delayed surgery, the demarcation of necrosis from vital tissue occurs resulting in less injuries to vital tissues. Therefore, in late surgery, there is less bleeding and the necrosectomy is more effective.

It is not known how long surgery can be postponed, if the patient can tolerate it, and will the longer delay result in more complications, such as increased rate of bowel fistulas or intestinal obstruction. If emergency surgery is needed earlier for other indications, such as abdominal compartment syndrome or bowel necrosis, drainage or necrosectomy is not routinely recommended [20, 97].

#### Statements (surgical strategy)

1. In infected pancreatic necrosis, percutaneous drainage as the first line of treatment (step-up approach) delays the surgical treatment to a more favorable time or even results in complete resolution of infection in 25–60% of patients and it is recommended as the first line of treatment (1A).
2. Minimally invasive surgical strategies, such as transgastric endoscopic necrosectomy or video-assisted retroperitoneal debridement (VARD), result in less postoperative new-onset organ failure but require more interventions (1B).
3. Considering mortality, there is insufficient evidence to support open surgical, mini-invasive, or endoscopic approach (1B).
4. In selected cases with walled-off necrosis and in patients with disconnected pancreatic duct, a single-stage surgical transgastric necrosectomy is an option (2C).
5. A multidisciplinary group of experts should individualize surgical treatment taking local expertize into account (2C)

##### Discussion

A systematic review of percutaneous catheter drainage as primary treatment for necrotizing pancreatitis consisted of 11 studies and 384 patients [97]. Infected necrosis was proven in 71% and 56% of patients did not require surgery after percutaneous drainage. In addition, percutaneous drainage allows delaying the later possible surgical intervention to a more favorable time.

An important question is what the preferred strategy is when percutaneous drainage does not result in resolution of the infection. The management options include open surgery, mini-invasive surgery, endoscopic surgery, and a combination of these. It is generally assumed that open surgery causes a more severe inflammatory response. There are various RCTs and a review comparing different strategies [104–106]. In summary, minimally invasive strategies (e.g., minimally invasive step-up approach, video-assisted retroperitoneal debridement, VARD, or endoscopic) result in less new-onset organ failure but require more interventions. However, no differences in mortality have been found. These conclusions are supported by a systematic review [107]. When interpreting the results, it should be noted that there is significant heterogeneity in patients, organ failures, and size as well as localization of necrosis. In addition, surgical techniques and indications for interventions are not uniform.

In a series of 178 selected cases with walled-off necrosis, 96% of the patients underwent a single-stage surgical transgastric necrosectomy with postoperative mortality and morbidity of 2% and 38%, respectively [108]. It is also a good option in patients with a disconnected duct syndrome.

When considering mortality, it is important to notice that pancreatitis-associated mortality is mostly not caused by infected necrosis. Therefore, in future studies, other outcomes measures should be considered. These outcome measures should be able to detect complete resolution of symptoms, quality of life, time to return to normal daily activities or work, and need for further interventions. Local expertize on different surgical approaches should be taken into account, since only a small percentage of patients require surgery and even in large centers the number of operations remains small. We recommend that a local multidisciplinary group of experts should individualize surgical strategy.

#### Statements (timing of cholecystectomy)

1. Laparoscopic cholecystectomy during index admission is recommended in mild acute gallstone pancreatitis (1A).
2. When ERCP and sphincterotomy are performed during the index admission, the risk for recurrent pancreatitis is diminished, but same admission cholecystectomy is still advised since there is an increased risk for other biliary complications (1B).
3. In acute gallstone pancreatitis with peripancreatic fluid collections, cholecystectomy should be deferred until fluid collections resolve or stabilize and acute inflammation ceases (2C).

##### Discussion

Two different systematic reviews state that index admission cholecystectomy for mild AGP is safe [109, 110]. In order to decrease the length of stay and the overall costs, cholecystectomy may be performed as early as the second hospital day, as long as the patient is clinically improving [111, 112]. Routine intraoperative cholangiography seems to be unnecessary in patients with mild gallstone pancreatitis and normalizing bilirubin levels [113]. If ERCP was performed during the index admission, the risk for recurrent biliary events, especially recurrent AGP, was diminished but still higher than same-admission cholecystectomy. A multicenter RCT with 266 patients concluded that interval cholecystectomy resulted in more gallstone-related complications, especially recurrent pancreatitis and colics, without increased cholecystectomy-related complications [114]. There is a single retrospective study of timing of cholecystectomy in patients with moderate to severe AGP with peripancreatic fluid collections [115]. This study reported more complications after early cholecystectomy.

### Open abdomen

Questions

1. Which are the indications for open abdomen in case of severe acute pancreatitis?
2. What is the best temporary abdominal closure system for open abdomen?
3. What is the correct timing for dressing changes?
4. What is the correct timing for abdominal closure?

#### Statements (open abdomen)

1. In patients with severe acute pancreatitis unresponsive to conservative management of IAH/ACS, surgical decompression and use of open abdomen are effective in treating the abdominal compartment syndrome (2C).
2. We suggest that clinicians should be cautious not to over-resuscitate patients with early SAP and measure intra-abdominal pressure regularly (1C).
3. We suggest that the open abdomen (OA) be avoided if other strategies can be used to mitigate or treat severe intra-abdominal hypertension in SAP (1C).
4. We recommend not to utilize the OA after necrosectomy for SAP (unless severe IAH mandates OA as a mandatory procedure) (1C).
5. We recommend not to debride or undertake early necrosectomy if forced to undertake an early OA due abdominal compartment syndrome or visceral ischemia (1A).

##### Discussion

The potential rationale for potentially utilizing OA management in severe acute pancreatitis (SAP) patients has historically been to potentially mitigate IAH/ACS, improve the drainage of inflammatory ascites, to allow potential pancreatic lavage, and to potentially allow easier relaparotomy with repeated necrosectomy [116–118].

However, in SAP, there is no level 1 evidence regarding the efficacy of the open abdomen for SAP, with no randomized controlled trials (RCTs) and no meta-analyses. There was a published protocol for such a study [119], but the reviewers could recover no evidence that this study was ever conducted.

As the next best level of evidence, there are existing consensus recommendations from the World Society of Emergency surgery [120], and the International Association of Pancreatology/American Pancreatic Association [20], that both recommend medical and minimally invasive management of severe intra-abdominal hypertension (IAH) leading to the abdominal compartment syndrome (ACS) as per the abdominal compartment syndrome management algorithms [101]. However, recognizing that established overt ACS is universally fatal if untreated, open decompressive laparotomy (DCL) will be required and is recommended if less invasive measures are not effective. When DCL is performed, the retroperitoneal cavity and the lesser omental sac should be left intact to reduce the risk of infecting peripancreatic and pancreatic necrosis [20, 121].

Related to this main recommendation, there are corollary statements that relate to the basic principles that over-zealous fluid resuscitation appears to be closely related to IAH/ACS occurrence in severe shock and that early necrosectomy is not warranted in SAP. A now classic study noted that early (< 72 h) versus late (> 12 days) necrosectomy had a 56% in early interventions to 27% in late operations, and the intraoperative blood loss was substantially reduced by a delayed approach, results that only continued to improve with continued refinements in surgery and critical care [122–124].

#### Statements (open abdomen management and temporary abdominal closure)

1. We recommend the use of negative pressure peritoneal therapy for OA management (1B).
2. We suggest fascial traction be added to NPWT methods (2B).
3. We suggest that further controlled studies be conducted on intra-peritoneal osmotic therapies in SAP (no recommendation)

##### Discussion

There were no RCTs or meta-analyses that directly presented comparative evidence regarding OA techniques in SAP, thus all evidence will be indirect related to the study of the OA in other related settings such as intra-peritoneal sepsis [125, 126], or mixed trauma-medical populations [127–130] with methodological concerns.

The study of Pliakos is notable because the randomized inclusion of fascial traction sutures in addition to peritoneal vacuum therapy was significantly associated with demonstrated superiority concerning a shorter open abdomen duration, reduced number of dressing changes, reduced re-exploration rate, higher successful abdominal closure rate, and reduced enteroatmospheric fistulae [125]. A RCT comparing active negative pressure peritoneal therapy versus more passive pressure demonstrated a mortality benefit with enhanced peritoneal pressure [129], corroborating non-randomized results [130], but a biological mechanism was not obvious. Several meta-analyses including non-randomized trial data have been conducted without clear superiority being demonstrated of any one method [131, 132]. The most contemporary of these did conclude “Although the best results in terms of achieving delayed fascial closure and risk of enteroatmospheric fistula were shown for NPWT with continuous fascial traction, the overall quality of the available evidence was poor, and uniform recommendations cannot be made” [131].

A final therapy to be carefully considered in OA management is that of direct peritoneal resuscitation (DPR), the intra-peritoneal instillation of dialysate fluid, which has been shown efficacious in trauma populations [133]. In a RCT from Smith and colleagues, intra-abdominal complications (8% vs. 18%), abscess rates (3% vs. 14%), and 30-day mortality were lower despite similar injury severity scores (13% vs. 28%; *p* = 0.06) (20). As there is no direct evidence in SAP patients, no recommendation was made concerning DPR.

#### Statement (timing of dressing changes)

1. Open abdomen re-exploration should be conducted no later than 24–48 h after the index and any subsequent operation, with the duration from the previous operation shortening with increasing degrees of patient non-improvement and hemodynamic instability (1C).

##### Discussion

There are no RCTs or meta-analyses concerning the timing of when a patient with an open abdomen should be taken back to the operating room specifically when the OA indication was SAP, nor for any other indication actually. Nor do other guidelines from recognized societies give evidence on when re-operation with an OA should take place [101, 131, 134, 135]. However, in one review, re-exploration performed more than 48 h after the initial operation resulted in a significantly higher mortality rate; and the lowest mortality rate (9%) was achieved in patients who underwent reoperation within 48 h [136].

Contemporary data indicate a linear correlation exists between days of OA and serious complications such as enterocutaneous fistula development [137]. Another prospective series noted that specifically, each hour delay in return to the operating room 24 h after initial laparotomy, and there was a 1.1% decrease in primary fascial closure, and a trend toward increased intra-abdominal complications after 48 h [138].

In the absence of any new data, the SAP OA reviewers suggest adopting the previous contemporary WSES OA management guidelines statement to maintain consistency across WSES sanctioned recommendations until new data warrants potential revisions [120]. As overall outcomes are markedly improved by avoiding early and un-necessitated pancreatic interventions [124], surgeons should resist any temptations to “mess with the pancreas” that might be presented in the course of a reoperation for the OA that would not be available in less complex cases of SAP.

#### Statements (timing for abdominal closure)

1. Early fascial and/or abdominal definitive closure should be the strategy for management of the open abdomen once any requirements for on-going resuscitation have ceased, the source control has been definitively reached, no concern regarding intestinal viability persist, no further surgical re-exploration is needed, and there are no concerns for abdominal compartment syndrome (1B).

##### Discussion

At the risk of possibly being considered facetious, the writing team emphasizes the importance of trying to optimize preventive strategies for IAH though careful and diligent resuscitation, early introduction of medical and minimally invasive management of IAH [101, 139, 140], to attempt to avoid progression to the ACS with a requirement for DCL.

Delayed fascial closure has been defined as formal fascial obtained seven or more days after the index OA procedure [141]. It has become apparent that complications are much higher and primary fascial closure much lower in those who undergo late versus early closure, although this may also be related to patient factors in uncontrolled non-randomized trials. Meta-analysis has however revealed that compared with delayed abdominal closure, early PFC was associated with reduced mortality and complication rate [142]. The former World Society of the Abdominal Compartment Syndrome thus recommended that among ICU patients with OAs, conscious and/or protocolized efforts be made to obtain early or at least same-hospital-stay abdominal fascial closure [101].

Similar to the preceding question, until new data regarding definitive OA closure in SAP or any other conditions becomes available, the reviewers suggest adopting the previous contemporary WSES management guidelines statement to maintain consistency across WSES sanctioned recommendations until new data warrants potential revisions [120].

## Conclusions

These guidelines present evidence-based international consensus statements on the management of severe acute pancreatitis from collaboration of a panel of experts. It contains 55 statements on diagnosis, management in the ICU, surgical and operative management, open abdomen, and antibiotic treatment. For some of the statements such as severity grading, imaging, use of prophylactic antibiotics and most aspect of the management in the ICU, the evidence is strong. For others, such as laboratory diagnostics and surgical strategies, for example, the evidence is quite weak requiring further studies. With accumulating knowledge, the statements need to be regularly updated.

## Abbreviations

ACS: Abdominal compartment syndrome
AGP: Acute gallstone pancreatitis
AKI: Acute kidney injury
ANC: Acute necrotic collection
AP: Acute pancreatitis
APACHE: Acute Physiology and Chronic Health Evaluation
BISAP: Bedside index of severity of acute pancreatitis
BMI: Body mass index
BUN: Blood urea nitrogen
CECT: Contrast-enhanced computed tomography
CRP: C-reactive protein
CT: Computed tomography
CTSI: CT severity index
DBC: Determinant-Based Classification of Acute Pancreatitis Severity
DCL: Decompressive laparotomy
DPR: Direct peritoneal resuscitation
ERCP: Endoscopic retrograde cholangiopancreatography
EUS: Endoscopic ultrasound
FNA: Fine-needle aspiration
GRADE: Grading of Recommendations Assessment, Development and Evaluation
HCT: Hematocrit
IAH: Intra-abdominal hypertension
IAP: Intra-abdominal pressure
ICU: Intensive care unit
LDH: Lactate dehydrogenase
MIC: Minimal inhibitory concentration
MRCP: Magnetic resonance cholangiopancreatography
MRI: Magnetic resonance imaging
NPWT: Negative pressure wound therapy
NS: Normal saline
NSAID: Non-steroidal anti-inflammatory drug
OA: Open abdomen
OF: Organ failure
PCA: Patient-controlled analgesia
PCT: Procalcitonin
POF: Persistent organ failure
PPV: Positive predictive value
RAC: Revised Atlanta Classification
RCT: Randomized controlled trial
RL: Ringer’s lactate
SAP: Severe acute pancreatitis
SAPS: Simplified Acute Physiology Score
SIRS: Systemic inflammatory response syndrome
SOFA: Sequential Organ Failure Assessment
TPN: Total parenteral nutrition
US: Ultrasound
VARD: Video-assisted retroperitoneal debridement
WBC: White blood cell
WON: Walled-off necrosis
WSES: World Society of Emergency Surgery
